# Reliability and Validity of a Temporal Distancing Emotion Regulation Task in Adolescence

**DOI:** 10.1037/emo0000744

**Published:** 2020-03-23

**Authors:** Chatrin Suksasilp, Sarah Griffiths, Catherine L. Sebastian, Courtenay Norbury, The SCALES Team

**Affiliations:** 1 Psychology and Language Sciences, University College London; 2 Department of Psychology, Royal Holloway, University of London; 3 Psychology and Language Sciences, University College London, and Department of Special Needs Education, University of Oslo

**Keywords:** emotion regulation, temporal distancing, adolescence, reliability, validity

## Abstract

Adopting a temporally distant perspective on stressors, also known as using a temporal distancing emotion regulation strategy, can alleviate distress. Young adults’ ability to adopt a temporal distancing strategy has previously been measured using an experimental temporal distancing task (Ahmed, Somerville, & Sebastian, 2018). In the current study, we evaluate the psychometric properties of this task in younger (*N* = 345, aged 10–11) and older (*N* = 99, aged 18–21) adolescents and explore developmental differences in the ability to use temporal distancing to alleviate distress. Participants listened to scenarios and rated negative affect when adopting a distant-future perspective, a near-future perspective, or when reacting naturally. We evaluated the test–retest reliability of the measure in older adolescents and its construct validity in both younger and older adolescents by assessing correlations with self-report measures of emotion regulation strategy use. Our findings broadly replicated those of Ahmed et al. (2018): Adopting distant- and near-future perspectives produced significantly lower self-reported distress relative to reacting naturally, with the distant-future strategy producing the least distress. Older adolescents alleviated their distress more effectively than younger adolescents and reported projecting further into the future. Regulation success scores on the temporal distancing task showed adequate test–retest reliability. However, these scores did not correlate with self-reported habitual use of temporal distancing or reappraisal strategies generally. These findings suggest that the ability to use a temporal distancing strategy for emotion regulation improves during adolescence, but that ability may not be related to habitual use of this strategy.

Adolescence is a period of heightened risk for social, emotional, and mental health problems, such as internalizing and externalizing concerns as well as psychopathology (e.g., Allen & Sheeber, 2008; Shortt, Stoolmiller, Smith-Shine, Mark Eddy, & Sheeber, 2010; Silk, Steinberg, & Morris, 2003). Three-quarters of mental health problems found in adulthood begin prior to the age of 18 (Murphy & Fonagy, 2012), and mental health problems in adolescence predict not only later mental health outcomes but also educational attainment, employment, and physical health in adulthood (Goodman, Joyce, & Smith, 2011; McLaughlin, Hatzenbuehler, Mennin, & Nolen-Hoeksema, 2011).

Adolescence is characterized by an increase in the frequency and intensity of emotionally challenging experiences. The ability to cope with these challenges is thought to depend on emotion regulation, the goal-oriented monitoring, evaluation, and modification of emotional reactions (Thompson, 1994). There is good evidence for an association between long-term mental well-being and individuals’ habitual use of cognitive strategies for emotion regulation, such as reappraisal, which involves reinterpreting emotional stimuli to change their emotional impact (e.g., Ayduk & Kross, 2010; Compas, Connor-Smith, Saltzman, Thomsen, & Wadsworth, 2001; Gross, 2002; Gross & John, 2003).

As such, a key goal for adolescent mental health research is to assess age-related changes in the effectiveness of emotion regulation. The “dual systems” and “imbalance” models of adolescent neurodevelopment propose that the increased risk for psychopathology and risk-taking during adolescence is brought about by the slow development of neural mechanisms supporting cognitive control relative to those that underlie reward-seeking and emotional reactivity (Casey, 2015; Shulman et al., 2016). Steinberg et al. (2018) demonstrated in a large multicultural study that reward-seeking peaks during adolescence at around age 19, whereas self-regulation continues to develop into the mid-20s. The imbalance between high affective-motivational responsiveness and lagging cognitive control is thought to underlie dysregulation of emotions in adolescence, leading to increased risk of mental illness (Casey et al., 2010; Lee et al., 2014; Paus, Keshavan, & Giedd, 2008). Emotion regulation is a cognitive control behavior that plays a protective role against mental illness (Troy, Wilhelm, Shallcross, & Mauss, 2010). Characterizing its developmental trajectory will advance our understanding of how risk for mental illness evolves across adolescence.

One type of emotion regulation strategy that has been linked to positive mental health outcomes is reappraisal (Ehring, Tuschen-Caffier, Schnülle, Fischer & Gross, 2010; Troy et al., 2010). Reappraisal is the process of cognitively reinterpreting emotional stimuli in order to achieve one’s goals. For example, psychological distancing, or self-distancing, is a reappraisal strategy that involves deliberately assuming a detached perspective on an emotional situation, which reduces the intensity and duration of negative affect compared to reflecting on negative events without strategy use (e.g., Ayduk & Kross, 2008; Kross & Ayduk, 2008; Kross, Ayduk, & Mischel, 2005; Verduyn, Van Mechelen, Kross, Chezzi, & Van Bever, 2012). Adolescents develop a larger repertoire of emotion regulation strategies as well as increased flexibility in applying the most adaptive strategies to different kinds of stressors (Zimmer-Gembeck & Skinner, 2011). Therefore, understanding the relative efficacy of different strategies at different ages is necessary for understanding the changes in emotion regulation that occur in development.

There is evidence that some types of reappraisal skill improve throughout adolescence. For example, McRae et al. (2012) examined developmental growth in regulation success using reappraisal strategies from early (10–13), middle (14–17), and late (18–22) adolescence. Participants were shown distressing images and instructed to reduce their distress using different reinterpretation strategies. McRae and colleagues found a linear increase in regulation success across adolescence but no change in emotional reactivity. Silvers et al. (2012) reported a similar developmental pattern when participants employed a physical self-distancing strategy to reduce distress, imagining themselves standing further away from the visual scene. Again, emotional reactivity was consistent across the age range, but age positively predicted regulation until age 18, at which point regulation success stabilized. Silvers et al. (2017) also found a linear association between age and physical self-distancing regulation success in participants aged 6–23. In contrast, Nook, Vidal Bustamante, Cho, and Somerville (2019) found no association between age and regulation success in 10- to 23-year-olds in a task where participants were presented with negative images and instructed to reappraise while speaking aloud about what they were thinking. Similarly, a recent study with a similar age group found no relationship between age and successful regulation using a temporal distancing strategy (Ahmed, Somerville, & Sebastian, 2018).

Temporal distancing is a self-distancing strategy that involves viewing a negative experience from a future time perspective. Reflecting on how one will feel “in the future” about a present real-world stressor reduces current negative affect (Bruehlman-Senecal & Ayduk, 2015), and greater habitual use of temporal distancing predicts fewer concurrent mental health symptoms above and beyond that predicted by broader reappraisal tendencies (Bruehlman-Senecal, Ayduk, & John, 2016). Ahmed et al. (2018) tested 12- to 22-year-olds in how successfully they could employ a temporal distancing strategy and did not find that age was related to “success” in downregulating emotion. Rather, temporal distancing success was high and stable across the age range, and the magnitude of distance adopted did not vary with age. One reason that Ahmed et al. (2018) may have failed to find an age difference in temporal distancing strategy use is that that they had few younger participants in the study. This may have led to a lack of power to detect age-related improvements in regulation success that have been found in other studies looking at reappraisal through self-distancing (McRae et al., 2012; Silvers et al., 2012, 2017).

Ahmed et al. (2018) tested temporal distancing success in adolescents in a controlled experimental paradigm. Participants rated their distress and arousal in response to negatively valenced scenarios (e.g., “You fail an important exam”), while being instructed to consider whether each situation would still affect them in the distant future, or in the near future, or to employ no strategy. Ahmed and colleagues found that adopting a distant-future perspective reduced arousal and distress ratings relative to adopting a near-future perspective or employing no strategy. Furthermore, individual differences in the magnitude of temporal distance projected while using the distant-future strategy were positively associated with the magnitude of distress and arousal reduction. In other words, not only did adopting a distant-future strategy reduce negative affect, but individuals who projected themselves further into the future benefited more from this strategy. However, these effects have yet to be replicated, and the reliability of the experimental task is unknown.

The current study therefore aimed to replicate Ahmed et al. (2018) by demonstrating that adopting a distant-future strategy downregulates negative affect and that the magnitude of downregulation is associated with how far into the future one projects. We test these hypotheses in both a group of older adolescents (aged 18–21 years; see Sawyer, Azzopardi, Wickremarathne, & Patton, 2018) recruited online and a group of younger adolescents (aged 10–11 years) tested individually. A second objective of the current study was to test for an improvement in temporal distancing success from early to late adolescence in a study with greater power to detect an age difference.

A final objective of this study was to evaluate the construct validity and test–retest reliability of the temporal distancing task. To test construct validity, we administered two self-report measures of emotion regulation. Ahmed et al. (2018) found no association between adolescents’ scores on the Emotion Regulation Questionnaire (ERQ), a measure of habitual reappraisal and suppression (Gross & John, 2003), and temporal distancing success. This null association may have been due to the task’s specific operationalization of temporal distancing, which differed from the broad conceptualization of reappraisal measured by the ERQ. In this study, we assessed the association between temporal distancing success and scores on the Emotion Regulation Questionnaire for Children and Adolescents (ERQ-CA), an adaptation of the ERQ for younger participants (Gullone & Taffe, 2012). We also tested the association between older adolescents’ temporal distancing success and scores on the Temporal Distancing Questionnaire (TDQ), a self-report measure of the habitual tendency to employ temporal distancing strategies (Bruehlman-Senecal et al., 2016), which may better tap into the underlying construct that the experimental task measures. We predicted that temporal distancing success scores would have small-to-medium positive associations (*r* = .1–.3) with ERQ-CA reappraisal scores and TDQ scores. Our analysis plan was preregistered on the Open Science Framework (osf.io/5rv2f; Suksasilp, Griffiths, Sebastian, & Norbury, 2019).

## Method

### Participants

#### Older adolescent sample

We aimed to recruit 100 participants aged 18–21 years to complete the study online, from the Prolific online recruitment platform and University College London (UCL) Psychology Divisional Subject Pool. All participants were invited to repeat the experimental task 7 days after initial assessment. Inclusion criteria were first-language speakers of English with no known language, reading, or mental health disorders. Participants were prescreened, and only those who met the inclusion criteria could access the experiment. All participants provided informed, written consent via the online experiment interface. UCL students received course credit for their participation, while Prolific participants received monetary payment at a minimum rate of 6 GBP per hour of participation.

#### Younger adolescent sample

Participants aged 10–11 were recruited as part of the Surrey Communication and Language in Education Study (SCALES), a longitudinal study of language and social, emotional, and behavioral development from ages 5 to 13 years (Norbury et al., 2016, 2017). Teachers completed assessment of language and behavior for 7,267 children who entered state-maintained school in the county of Surrey in the United Kingdom in September 2011. Stratified random sampling was used to select a cohort of 636 children representing the full range of language ability (see Norbury et al., 2016, for details). Children attending special schools for children with severe intellectual or physical disability and children with English as a second language were excluded. Children were invited to take part in in-depth assessments in Year 1 (T2, ages 5–6 years, *N* = 529), Year 3 (T3, ages 7–8 years, *N* = 499), and Year 6 (T4, ages 10–11 years, *N* = 385). The T4 assessment battery included the temporal distancing task and the ERQ. The total length of the test battery was approximately 2 hr. Testing took place during the school day. Consent procedures and study protocol were developed in consultation with Surrey County Council and approved by the UCL Research Ethics Committee (9733/002). Informed consent was collected from parents before in-depth assessments at T2 and T4. Informed assent was collected from children prior to T4 assessment. Children were given certificates and small prizes at the end of each assessment session. The study was approved by the UCL Research Ethics Committee (Project ID 9733/002).

### Measures

#### Temporal distancing task

Participants listened to stimuli consisting of short sentences spoken by a female speaker of Southern British English describing either negatively valenced scenarios (*N* = 30; e.g., “You fail an important exam”) or neutral scenarios (*N* = 10; e.g., “The main hall is being repainted”). The negative stimuli were sorted into three sets of 10 negative scenarios, which Ahmed et al. (2018) matched for ratings of valence, arousal, and the duration of emotional impact, as well as type of stressor and social content. Each of the three sets of negative scenarios was randomized to one of the three strategy conditions: (a) adopting a distant-future perspective, (b) adopting a near-future perspective, or (c) no distance condition. The four sets of scenarios (one neutral and three negative) were presented in a fixed order with the neutral set always presented first. The neutral set was always paired with the no distance instruction. In total, there were four conditions: distant-future negative, near-future negative, no distance negative, and no distance neutral. Participants completed 10 trials for each condition split into two blocks of five trials. The two blocks for each condition were presented consecutively, and scenarios were randomized within each block. The no distance neutral block was always completed first, followed by the no distance negative, near-future negative, and distant-future negative blocks in a random order.^1^

In each trial, the participant heard the scenario and saw the written instruction “imagine this happened to you today.” After this, the screen displayed the instruction “Imagine how this would affect you XX,” where XX was replaced by (a) “NOW” in the no distance neutral and no distance negative conditions, (b) “NEXT WEEK” in the near-future negative condition, or (c) “MANY YEARS FROM NOW” in the distant-future negative condition. Participants then pressed the “Next” button to advance to the final screen, which displayed the question “How do you feel right now?” Participants responded by clicking on one of nine cartoon faces (ranging from unhappy to happy with a neutral midpoint). There was no time limit for making a response or clicking on the “Next” button. Every five trials participants were allowed a break with no time limit before pressing the “Next” button to continue.

Before starting the task, participants read the task instructions and completed four practice trials. At the end of the task, participants reported the distance in time they adopted on distant-future trials, ranging from “one year from now” to “nine years or more from now” in 1-year increments.

This procedure deviated from Ahmed et al.’s (2018) procedure in a number of ways in order to make it more suitable for younger participants. First, scenarios were presented orally, rather than in writing, to make it easier for younger children who may not be fluent readers. Second, Ahmed et al. (2018) asked participants to rate both distress (“How upset do you feel right now?”) and arousal (“How anxious/stressed do you feel right now?”), while we combined these two responses into one, worded as “How do you feel right now?” Third, response options were nine schematic faces ranging from frowning to smiling, rather than numbers. Finally, we asked participants to rate distance in time adopted for the distant-future condition only once at the end of the experiment, while Ahmed et al. (2018) asked this question after every distant-future and near-future trial. These changes were necessary to reduce the length of the experimental session and maintain engagement of younger participants.

#### ERQ-CA

Both older and younger adolescents completed the ERQ-CA (Gullone & Taffe, 2012) to measure habitual reappraisal. The ERQ-CA is a revision of Gross and John’s (2003) ERQ that is designed to be more accessible to younger populations. The ERQ-CA includes a six-item reappraisal scale containing items such as “when I want to feel happier, I think about something different,” to which participants rate their agreement on a 5-point scale (1 = *strongly disagree,* 2 = *disagree,* 3 = *half and half*, 4 = *agree*, 5 = *strongly agree*). The test–retest reliability was found to be moderate over a 12-month period, intraclass correlation *r* = .37–.47 (Gullone & Taffe, 2012). Internal consistency in the current study was moderate, with Cronbach’s alpha for the ERQ-CA reappraisal scale ranging from .63 for younger adolescents to .66 for the older adolescents.

#### TDQ

The older adolescents completed the TDQ (Bruehlman-Senecal et al., 2016) to measure habitual temporal distancing. The TDQ contains eight items, such as “I focus on how my feelings about the event may change with time,” to which participants rate their agreement on a 7-point scale (1 = *strongly disagree* to 7 = *strongly agree*). Exploratory factor analysis has shown that the TDQ measures one latent factor that is separate from the reappraisal factor in the ERQ (Bruehlman-Senecal et al., 2016). Stability of the TDQ over a 1-month period was estimated to be good, intraclass correlation *r* = .63 (Bruehlman-Senecal et al., 2016), and internal consistency in the current study was moderate (Cronbach’s α = .72).

#### Receptive One Word Picture Vocabulary Test

Both older and younger adolescents completed the Receptive One Word Picture Vocabulary Test (ROWPVT-4; Martin & Brownell, 2010), a standardized assessment of receptive vocabulary, in an online format. The test involves selecting a picture from four options to match a word spoken. Words were audio recordings spoken by a female voice with a southern British accent. We calculated standard scores according to the manual to provide an index of verbal IQ for each participant.

### Data Analysis

The analysis plan for this study was preregistered on the Open Science Framework (https://doi.org/10.17605/OSF.IO/R9CT5). Any deviations from this plan are noted in the Results section. All analyses were conducted in R (R Core Team, 2019), and the R markdown script to produce the results reported below is available on the Open Science Framework (https://osf.io/5rv2f). Data are also available on the Open Science Framework, but note that data from 10 participants in the younger adolescent group are not included in this open data set, as their parents did not provide consent for open data sharing. Therefore, the results from analyses that include the younger adolescents conducted with the open data set will differ slightly from the results reported below.

#### Sample size justification

Our younger adolescent sample size was determined by the size of the cohort and rate of attrition. To determine the number of older adolescents to recruit, we conducted an a priori power analysis to determine the number of participants required to replicate the main effect of temporal distance on distress from Ahmed et al. (2018). Using G*Power 3.1 (Faul, Erdfelder, Lang, & Buchner, 2007), we estimated that a sample of nine participants would provide 95% power to detect a difference in distress ratings of partial η^2^ = .27 in a repeated-measures analysis of variance (ANOVA) with three levels of distance. However, because we were also interested in age group difference between the younger and older adolescents, we decided to recruit 100 older adolescents to make the sample sizes more equal. To determine the number of older adolescents to recruit for retest, we used a simulated estimate of the sample size required to achieve a 95% confidence interval width of .3 for the reliability interclass correlation (ICC), assuming the task had a true reliability ICC of .7 (Doros & Lew, 2010). The required sample size for this ICC and level of precision was 50.

Given our younger adolescent sample size was fixed, we ran sensitivity analyses rather than a priori power calculations to determine the effect size we would be able to detect given an anticipated sample size of 399 younger adolescents and 100 older adolescents. Sensitivity analysis in G*Power 3.1 (Faul et al., 2007) indicated that we would have 95% power to detect small-to-medium (*d* = .40 or greater) differences between the two age groups distancing success. For associations between temporal distancing success and (a) distance in time adopted, (b) ERQ-CA score, and (c) TDQ score, sensitivity analyses indicated we would have 95% power to detect medium (*r* = .34 or greater) associations within the older adolescent group and small (*r* = .18 or greater) associations within the younger adolescent group.

#### Reactivity manipulation check

Before conducting any analysis, we calculated emotional reactivity scores for each participant by taking average distress rating for no distance neutral trials from average distress rating for no distance negative trials. As planned, we excluded adolescents if they had a negative reactivity score. Two of the younger adolescents had negative emotional reactivity scores (indicating more distress after neutral scenarios than negative scenarios) and were therefore excluded from further analysis. Emotional reactivity scores did not differ between the two age groups, *F*(1, 439) = 2.43, *p* = .12, η^2^ < 0.01.

## Results

### Participants

#### Older adolescents

A total of 108 older adolescents were recruited: 4 did not complete the study in time, 2 reported technical failure, and 3 reported their age to be above 21 years. Of the remaining 99 participants, 5 were recruited from the UCL Psychology Divisional Subject Pool and the rest were recruited from Prolific. Demographic information is presented in Table 1. Fifty-seven participants (mean age = 20.20 years, 26 female) completed the retest session 1 week later. These participants did not differ from the participants who only took part at T1 on age, *t*(85) = 1.34, *p* = .19, *d* = 0.27; verbal IQ, *t*(78) = −0.50, *p* = .62, *d* = −0.10; or temporal distancing success at T1, *t*(89) = 0.04, *p* = .97, *d* < .01.[Table-anchor tbl1]

#### Younger adolescents

Of the 384 participants assessed for the SCALES T4 assessment battery, 344 (90% of total sample) completed the temporal distancing task. Reasons for not completing the task included time constraints, technical difficulties, and/or language/cognitive problems that impeded compliance with task instructions. As noted, 2 additional participants were excluded due to negative emotional reactivity, leaving a total sample of 342 younger adolescent participants for analysis.

Demographic information is presented in Table 1. The younger and older adolescent groups both had a wide range of verbal IQ scores with a mean value above the population average. However, the verbal IQ of the younger adolescent group was slightly lower on average than the older adolescent group, *t*(152) = −2.88, *p* = .005, *d* = −0.33. We therefore repeat analyses comparing the two age groups with verbal IQ scores included as a predictor, when verbal IQ was associated with the outcome, to account for this difference in verbal ability.

### Hypothesis 1: Replicating the Temporal Distancing Effect

**Figure 1 fig1:**
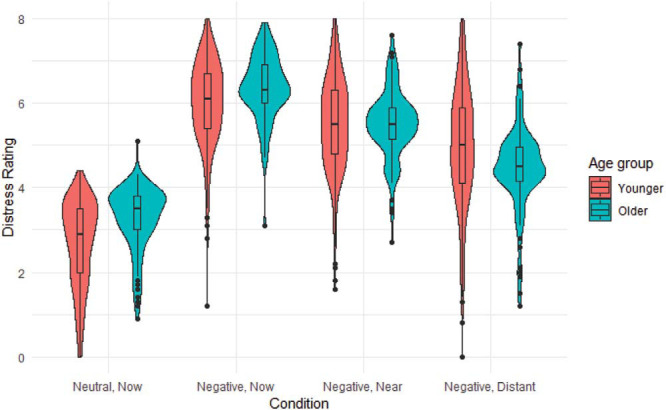
Violin plots with box plot for distress ratings in each condition by age group.

Our first hypothesis was that adopting a distant-future perspective would reduce distress relative to no distance in both younger and older adolescents. Figure 1 shows the mean distress rating in each condition for each age group. A 2 group × 3 condition ANOVA comparing the three negative conditions confirmed a main effect of condition, *F*(2, 907) = 242.62, *p* < .001, η^2^ = .33, and an interaction between condition and group, *F*(2, 907) = 19.09, *p* < .001, η^2^ = .03, but no main effect of group, *F*(1, 1176) = 0.64, *p* = .42, η^2^ = .001. Tukey’s honestly significant difference post hoc tests for each group separately indicated that both younger and older adolescents reported greater distress in the no distance negative than in the near-future negative condition (younger adolescents, 0.53 ± 0.07, *p* < .001; older adolescents, 0.87 ± 0.96, *p* < .001) and greater distress in the near-future negative than distant-future negative condition (younger adolescents, 0.54 ± 0.07, *p* < .001; older adolescents, 1.04 ± 0.96, *p* < .001). This supports our first hypothesis that projecting into the distant-future reduces self-reported distress in both older and younger adolescents, relative to not using a temporal distancing strategy and even relative to a projecting into the near future.[Fig-anchor fig1]

### Hypothesis 2: Age Differences in Temporal Distancing Success

Our second hypothesis was that the older group would be more successful at regulating their emotion than the younger group. The significant interaction in the group by condition ANOVA reported above is consistent with this hypothesis, as it may be driven by a smaller reduction in distress from the no distance negative condition to the distant-future negative condition for the younger adolescents compared to older adolescents. To test this statistically, we calculated regulation success scores using the same method as Ahmed et al. (2018) by taking distress in distant-future negative trials from distress in no distance negative trials. A one-way ANOVA comparing age groups on these difference scores provided evidence for a group difference in distancing success, *F*(1, 439) = 26.89, *p* < .001, η^2^ = .06, with older adults showing a greater reduction in distress from no distance negative trials to distant-future negative trials relative to younger adolescents. When verbal IQ scores were included as a predictor, there was a small association between verbal IQ and success, *F*(1, 439) = 4.11, *p* = .04, η^2^ = .008, but the association between age and success did not attenuate, *F*(1, 439) = 27.48, *p* < .001, η^2^ = .06, and there was no interaction between age and verbal IQ, *F*(1, 439) = 0.60, *p* = .44, η^2^ = .001.

We conducted an additional exploratory analysis to see if older adolescents also showed a greater reduction in distress from the near-future condition to the distant-future condition in comparison to the young adolescents. Such an analysis would provide further evidence that distance matters and that nuanced future thinking improves with age. To do this, we calculated difference scores by taking distress in distant-future negative trials from distress in near-future negative trials. A one-way ANOVA comparing age groups on these scores provided further evidence for a group difference in distancing success, *F*(1, 439) = 16.64, *p* < .001, η^2^ = .04, with older adolescents showing a greater reduction in distress from near-future negative trials to distant-future negative trials (*M* = 1.04, *SD* = 0.94) relative to younger adolescents (*M* = 0.54, *SD* = 1.10). When verbal IQ scores were included as a predictor, the association between age on success remained, *F*(1, 439) = 16.69, *p* < .001, η^2^ = .04, and there was no main effect of verbal IQ or interaction between verbal IQ and age (*p*s > .64, η^2^ < .001).

### Hypothesis 3: Age Differences in Distance Projected

**Figure 2 fig2:**
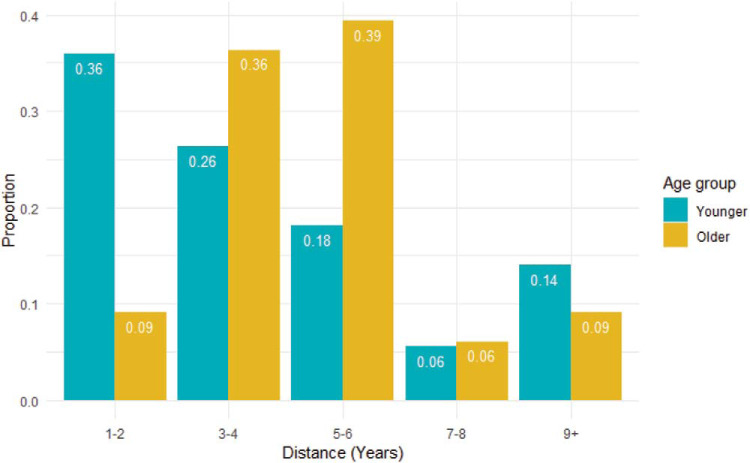
The reported distance projected into the future (in years) for each age group as a proportion of the responses from that age group. Distances are grouped into bins of 1–2 years, 3–4 years, 6–7 years, 8–9 years, and 9 or more years.

Our third hypothesis was that (a) older adolescents would project a greater distance into the future than younger adolescents and (b) distance projected would be positively associated with regulation success. To test Hypothesis 3a, we had planned to analyze individual differences in distance projected using a one-way ANOVA as in Ahmed et al. (2018). However, because we only measured distance projected once at the end of the experiment, rather than after every near-future and distant-future trial, the data were ordinal, so a one-way ANOVA was inappropriate. Instead, we conducted an exploratory analysis in which we grouped participants into five groups based on the distance they projected: 1–2 years, 3–4 years, 5–6 years, 7–8 years, and 9 or more years. Figure 2 illustrates the proportion of participants in each age group for each distance-projected bin. A Wilcoxon Mann–Whitney test provided evidence for a small group difference in distance projected, driven by older adolescents projecting further into the future than younger adolescents, *W* = 20,686, *p* < .001, Vargha and Delaney A = .61. Verbal IQ scores were not associated with distance projected, *r*_s_(438) = .04, *p* = .36.[Fig-anchor fig2]

Spearman correlations were run to assess association between distance and success due to the ordinal nature of the distance variable. Contrary to Hypothesis 3b, there was no evidence for an association between distance and regulation success in either the younger adolescents, *r*_s_ (339) = .01, *p* = .84, or older adolescents, *r*_s_ (97) = .13, *p* = .20.

### Hypothesis 4: Test–Retest Reliability

Our fourth hypothesis was that the temporal distancing task would yield acceptable levels of reliability (e.g., an ICC above .50; Koo & Li, 2016).^2^ We calculated the intraclass correlation between temporal distancing success scores from measurements taken 1 week apart in the older adolescent group. It was not possible to collect retest data for the younger adolescent group since we only visited each child once as part of a larger study. Based on a single-rater, two-way mixed-effects model, the absolute agreement ICC was .59, 95% CI [.39, .73]. This indicates that the temporal distancing success measure had acceptable test–retest reliability (Koo & Li, 2016).

We conducted exploratory analysis on the retest data to provide further evidence of replication of the temporal distancing effect. The main effect of condition was replicated at T2, *F*(2, 112) = 103, *p* < .001, η^2^ = .61, as were all pairwise comparisons (*p*s < .001). We additionally conducted an exploratory analysis comparing success scores at T1 to success scores at T2 to determine whether there was a practice effect. There was no increase in temporal distancing success scores from T1 to T2, *t*(56) = 0.80, *p* = .42, *d* = 0.12, suggesting there was no practice effect.

### Hypothesis 5: Association With Self-Report Measures

Our fifth hypothesis tested the validity of the temporal distancing task by assessing the correlations between temporal distancing success score and (a) ERQ-CA reappraisal score (both age groups) and (b) TDQ total score (older adolescents only). We found no evidence for an association between the temporal distancing success score and reappraisal score from the ERQ in either younger adolescents, *r*(336) = −.09, *p* = .10, or older adolescents, *r*(97) = −.12, *p* = .25. We also found no association between temporal distancing success score and total score on the temporal distancing questionnaire in older adolescents, *r*(97) = .15, *p* = .15.

We conducted an additional exploratory analysis to determine whether distance projected was associated with self-reported habitual reappraisal and temporal distancing. We found no association between distance projected and ERQ reappraisal scores in either younger adolescents, *r*(335) = .04, *p* = .42, or older adolescents, *r*(97) = −.09, *p* = .38, nor was there an association between temporal distancing success score and total score on the temporal distancing questionnaire in older adolescents, *r*(97) = .04, *p* = .66.

### Exploratory Analyses: Age Differences on the ERQ

To gain further insight into age differences in reappraisal, we conducted exploratory analyses to compare older and younger adolescents on self-reported reappraisal score from the ERQ. Younger adolescents reported greater use of reappraisal compared to older adolescents, *t*(171) = 21.71, *p* < .001, *d* = 2.37.

## Discussion

The present study investigated differences in temporal distancing skill between early and late adolescence using a cross-sectional design and evaluated the reliability and validity of an experimental temporal distancing task. In doing so, this study replicated the temporal distancing effect found by Ahmed et al. (2018) in both age groups. However, older adolescents downregulated negative affect more successfully than younger adolescents and projected further into the future. The test–retest reliability of the experimental task was within acceptable limits; however, scores on the task were not meaningfully associated with self-report measures of habitual use of either emotion reappraisal generally or temporal distancing specifically in either age group.

### Temporal Distancing Attenuates Ratings of Distress

This study confirmed that temporal distancing can be an effective emotion regulation strategy when presented with hypothetical but age-appropriate and moderately distressing scenarios. In addition, adopting a distant-future perspective was more effective at alleviating distress than adopting a near-future perspective, in line with Ahmed et al. (2018) and Bruehlman-Senecal and Ayduk (2015). This finding suggests that temporal extent is a key factor in temporal distancing efficacy as opposed to more general cognitive processes associated with imagining any future time point (although we acknowledge that the present study is unable to elucidate the precise mechanism by which distance exerts its salutary effects).

That said, while Ahmed et al. (2018) found a positive correlation between individual differences in distance projected and individual differences in regulation success during distant-future negative trials, this study found no such association. In the current study, we did not find a meaningful association between the distance individuals reported that they projected on distant-future trials and regulation success in either the older or younger adolescent group. This finding appears to undermine the idea that distance projected impacts temporal distancing efficacy; however, alternative explanations likely account for the discrepancy between our finding and Ahmed et al. First, the two studies differed in how participants reported distance projected in distant-future trials. In Ahmed et al. (2018), participants rated the approximate distance in time projected immediately following each trial, whereas participants in the current study gave only one rating at the end of the experiment. This deviation from Ahmed et al.’s procedure was made to shorten the procedure and make it more accessible for younger participants; however, it may have compromised the accuracy of the current study’s distance projected measure. Participants may have projected to different times in the future for different stimulus items in the distant-future condition, and this variation in distance projected is well captured when participants give a rating after seeing each item. However, when participants are required to give a single retrospective rating, as in the present study, they are unlikely to recall accurately how far on average into the future they projected across all trials. The lack of precision of the present study’s measure of individual differences in distance projected likely limited the power to detect a meaningful association between distance projected and success.

A second explanation for the lack of association between individual differences in distance projected and regulation success likely stems from the statistical limitations of experimental tasks in accurately ranking individuals in the underlying skill (Hedge, Powell, & Sumner, 2018). Experimental cognitive tasks are designed to produce robust or “reliable” effects of treatment or condition that nearly always replicate by minimizing between-participants variance (in this case, the within-subject effect of distance on distress). When the dependent variable is a difference score, as in the temporal distancing task, the between-subjects variance is further limited. As a result, the size of correlations between experimental task scores and other measures, such as the distance projected rating (Spearman, 1910), is reduced. Note that although poor reliability leads to inconsistency in ranking individuals within a group, which is problematic for correlation, it does not mean that between-group differences, such as the age group difference identified in this study, cannot be detected (Hedge et al., 2018).

A combination of these two methodological issues is likely to account for the lack of evidence for an association between distance projected and success scores in the current study. As such, the findings of this study should not be interpreted as a challenge to the view that distance matters for successful temporal distancing, particularly given the results of our group comparison, discussed below.

### Older Adolescents Experience Greater Temporal Distancing Success and Can Imagine a More Distant Future

As hypothesized, the older adolescent group more successfully downregulated their emotion using temporal distancing than the younger group and reported projecting further into the future when doing so. This age difference in regulation success could be explained neither by an age difference in emotional reactivity, which was equal across the two groups, nor by age differences in self-report of affect, as there was no significant main effect of age on affect ratings. This finding suggests that older adolescents are more proficient at using the temporal distancing as an emotion regulation strategy to alleviate distress, potentially because they project further ahead in time while doing so.

Our findings are consistent with previous cross-sectional studies of reappraisal and self-distancing success in adolescence, which suggest that while young adolescents have the ability to regulate their emotions, they do so less effectively than older adolescents (McRae, Gross, et al., 2012; Silvers et al., 2012, 2017). The present study extends this developmental pattern to temporal distancing by demonstrating that although 10- and 11-year-olds are capable of alleviating distress with temporal distancing, this ability continues to improve throughout adolescence. However, our findings do contrast with the findings of Ahmed et al. (2018), who did not find a correlation between age and temporal distancing success in 12- and 22-year-olds. This discrepancy is likely explained by the greater statistical power in the current study to analyze potential age effects.

There are multiple potential explanations for age-related improvements in temporal distancing success. First, temporal distancing may simply improve in line with developmental improvements in general reappraisal ability. Second, temporal distancing is thought to reduce distress by activating one’s “impermanence focus,” awareness of the impermanence of stressors and emotional reactions (Bruehlman-Senecal & Ayduk, 2015), which may improve with age. Bruehlman-Senecal and Ayduk (2015) demonstrated that self-reported “impermanence focus” (e.g., “I told myself that my feelings about the problem are temporary”) consistently mediated the link between temporal distancing and reduced distress. Furthermore, experimentally manipulating participants’ focus on the impermanence of a current real-life stressor reduced negative associated emotions. Although research into mindfulness has produced a self-report measure of impermanence focus, referred to as “decentering” (Fresco et al., 2007), no study to our knowledge has investigated the development of impermanence focus across adolescence. We speculate that impermanence focus improves across adolescence, leading to improved temporal distancing; as older adolescents have had more life experience than younger peers, they are likely to remember negative events or emotional reactions that subsided with time. Furthermore, adolescent neurodevelopment includes maturation of interactions between the hippocampal memory and prefrontal control systems, which is thought to allow older adolescents to better draw upon prior experiences to support goal-directed behaviors such as emotion regulation (Murty, Calabro, & Luna, 2016). Younger adolescents, who have fewer prior experiences and less capacity to integrate them with active attempts at emotion regulation, may struggle with impermanence focus, hindering the efficacy of temporal distancing.

Another explanation for older adolescents’ greater temporal distancing success may lie in their increased capacity for episodic future thinking, or “mental time travel.” Episodic future thinking, the ability to simulate and “preexperience” future events (Tulving, 1985), develops during adolescence (Abram, Picard, Navarro, & Piolino, 2014). Gott and Lah (2014) found that 14- to 17-year-olds generated future episodic events in more detail compared to 8- to 11-year-olds, indicating developmental growth in the ability to simulate future events. Abram et al. (2014) extended this finding by observing that difficulties generating future imagined events persist until age 12, with a gradual improvement in future thinking from ages 6–8 to age 21.

Episodic future thinking is a necessary skill for individuals to consider how they might feel in the near or distant future about a current stressor. The act of simulating and preexperiencing a future event is suggested to rely on the recombination of past event details into a single occurrence (Schacter & Addis, 2007). As such, the range of future events that younger adolescents can simulate may be constrained by their knowledge base or episodic and semantic memories used to simulate “what it would be like” to be older. Limitations in episodic future thinking would clearly impact performance on the temporal distancing task used here. Our participants (aged 10–11) were younger than the youngest participants in Ahmed et al.’s (2018) study (minimum age 12). It may be that by age 12, adolescents’ future thinking may have been sufficiently developed for effective use of temporal distancing to alleviate distress. If so, the inclusion of younger children in the current study would explain the difference in findings between the two studies.

Our data also allowed us to compare the two age groups on self-reported use of reappraisal. Perhaps surprisingly, since our own and other studies have suggested an increase in success in reappraisal (McRae, Gross, et al., 2012; Silvers et al., 2012, 2017), we found that younger adolescents reported using reappraisal more often than older adolescents. However, this finding is consistent with a previous study that compared two groups of adolescents on the ERQ and also found age to be negatively associated with self-reported reappraisal (Gullone, Hughes, King, & Tonge, 2010). These results may suggest that younger adolescents try to use reappraisal more often but are less successful at using it to regulate emotion than older adolescents. Alternatively, it may be that they are less accurate at reporting their habitual strategy use. Nook et al. (2019) measured tendency to use particular regulation strategies in an experimental task where participants were instructed to speak aloud about how they were regulating. In this task, which directly measured strategy use rather than relying on self-report, age was associated with an increase in some types of reappraisal, such as self-distancing, but a decrease in other types of reappraisal, such as changing consequences. Future studies should compare experimental measures of different reappraisal strategy use and effectiveness with self-report of habitual strategy use.

### This Task Is Reasonably Reliable, but Performance Is Not Associated With Self-Reported Habitual Strategy Use

Conclusions about the utility and development of temporal distancing as a strategy for emotion regulation depend critically on the reliability and validity of the task. The current study is the first to report test–retest reliability of this task, and the intraclass correlation coefficient surpassed the threshold for acceptable reliability (ICC > .50; Koo & Li, 2016). Although the number of older adolescent participants who completed the retest phase limited the precision of reliability estimates, the 95% confidence interval for both the absolute and consistency ICCs ranged from “fair” to “substantial” (Landis & Koch, 1977). This result indicates that the temporal distancing task is an adequate instrument for assessing the longitudinal development of emotion regulation success.

However, the validity of the task as a measure of emotion regulation was less clear, as the experimental task did not correlate with self-reported scores on measures of habitual reappraisal and habitual temporal distancing. These results are consistent with Ahmed et al.’s (2018) finding that temporal distancing success was not associated with the ERQ, a self-report of habitual reappraisal. The current study extends this work to the TDQ, which focuses explicitly on habitual temporal distancing and is therefore more closely aligned to the construct that the experimental procedure seeks to tap. These findings contrast with studies reporting a positive correlation between scores on the ERQ and an experimental measure of reappraisal success (McRae, Jacobs, Ray, John, & Gross, 2012).

One likely possibility is that the temporal distancing task and the TDQ measure different aspects of an individual’s temporal distancing competencies (see Silvers & Guassi Moreira, 2019). The temporal distancing task measures the extent to which one can alleviate distress when trained and instructed to employ this strategy; in other words, it taps one’s capacity for temporal distancing. The TDQ, on the other hand, measures how often one employs temporal distancing strategies spontaneously, or one’s tendency to use the strategy in everyday contexts as required. Thus, the temporal distancing task and the TDQ may measure psychological constructs that do not naturally correlate with one another.

A second possibility stems from statistical limitations of experimental cognitive tasks, such as the temporal distancing task, in reliably ranking individuals (Hedge et al., 2018). This issue is compounded by the reliability of the self-report measures, as both the ERQ-CA reappraisal scale (ICC = .37–.47) and the TDQ (ICC = .63) have comparable (adequate but not excellent) test–retest reliability to the temporal distancing task (Bruehlman-Senecal et al., 2016; Gullone & Taffe, 2012). As such, power to detect a significant association between success scores on the temporal distancing task and either self-report measure was likely limited by the reliabilities of the individual measures.

### Limitations and Future Research

In the current study, we used a controlled experimental paradigm to study the effect of temporal distancing on adolescents’ distress levels. The findings demonstrate that adolescents can use a temporal distancing strategy to alleviate distress in a controlled setting. However, this does not necessarily mean they can use a temporal distancing strategy to alleviate distress in their everyday lives when experiencing intense emotions and without explicit instruction. Future studies should test whether adolescents benefit from being trained to use temporal distancing to deal with real-life stressors, such as exam anxiety, and look at whether the benefit of such training is related to the ability to employ temporal distancing in controlled settings. If success on this task relates to training effects, it would suggest that the task measures one’s potential to benefit from using a temporal distancing strategy in the real world.

The cross-sectional design employed in this study provides indirect rather than direct evidence of developmental gains in temporal distancing. The characteristics of the two samples may differ due to different sampling techniques, as the younger adolescent sample was a population cohort sample, while the older adolescent sample included self-selected members of the Prolific community and students. We opted to recruit older adolescents from Prolific in order to have a more representative sample of young people than would be seen if recruitment were limited to university undergraduates. Nevertheless, it remains possible that age differences were actually due to other, unmeasured differences between the samples (e.g., mental health conditions or general cognitive ability). However, including verbal IQ as a predictor of temporal distancing success revealed that although verbal IQ was related to success, it did not account for the age group difference, suggesting that group differences in general cognitive ability (at least in the verbal domain) were not responsible for age group differences.

Additionally, the testing conditions were different between the two groups, as the older adolescents completed the task online, while the younger adolescents completed the task with an experimenter along with other assessments. There is good evidence that results from online and in-lab testing are comparable (Clifford & Jerit, 2014; Crump, McDonnell, & Gureckis, 2013); however, it may be that the younger sample was more fatigued than the older adolescent sample given they completed additional assessment. Nevertheless, both groups demonstrated the temporal distancing effect, and the groups did not differ in their reactivity to the negative scenarios. If one group was more fatigued than another, we would expect them to be less accurate at reporting their emotional response across all trials, in which case they would show reduced reactivity as well as reduced success. The fact that the groups showed equal reactivity therefore suggests that testing conditions did not fundamentally change how participants reacted to the stimuli. Future research should employ longitudinal designs in order to characterize adolescent growth in temporal distancing skill.

Future research could also investigate the role of language proficiency in enabling both episodic future thinking and successful temporal distancing. Cognitive emotion regulation strategies like reappraisal and temporal distancing involve a high language load, and use of language is associated with emotion regulation success in adults (Nook, Schleider, & Somerville, 2017). Language competence also continues to develop throughout adolescence and may contribute to poorer temporal distancing performance in younger adolescents.

## Conclusion

Temporal distancing is an effective strategy for emotion regulation for both younger and older adolescents, although the latter demonstrate greater success in using it to alleviate distress. This study is the first to report the test–retest reliability of Ahmed et al.’s (2018) temporal distancing task, which indicates that the task has adequate reliability. As such, it may be a useful tool for characterizing longitudinal growth in capacity for emotion regulation. However, experimental tasks of temporal distancing may not index an individual’s tendency to employ emotion regulation strategies in everyday situations that require them. Different tasks are therefore needed to provide converging evidence to elucidate the relationship between emotion regulation and the onset of mental health conditions in adolescents.

## Figures and Tables

**Table 1 tbl1:** Demographic Information and Descriptive Statistics for Each Age Group

Variable	Younger adolescents (*N* = 342)	Older adolescents (*N* = 99)
Female, *n* (%)	175 (50.87)	50 (50.51)
Age, *M* (*SD*)	11.15 (0.34)	20.07 (1.07)
Verbal IQ, *M* (*SD*)	105.16 (16.38)	110.77 (17.29)
Reactivity, *M* (*SD*)	3.32 (1.41)	3.10 (1.17)
Success, *M* (*SD*)	1.07 (1.48)	1.91 (1.12)
Distance, *M* (*SD*)	4.04 (2.63)	4.64 (2.05)
ERQ reappraisal, *M* (*SD*)	20.94 (3.55)	12.68 (3.27)
TDQ, *M* (*SD*)		35.24 (6.72)
*Note*. ERQ = Emotion Regulation Questionnaire; TDQ = Temporal Distancing Questionnaire; IQ = Intelligence Quotient.		

## Footnotes

1 This procedure differed slightly from the original study due to a programming error. In the original study, participants completed two runs of four blocks of five trials, one block from each condition in each run. Within each run, conditions were presented in a random order and the order of scenarios was randomized within the blocks. In our study, participants completed a single run of eight blocks. The order of the blocks of scenarios was always the same, with the two neutral blocks presented first. The order of the instructions that were paired with the negative blocks (now, near future, distant future) was randomized (so the negative conditions were randomized) and the two blocks in each condition were consecutive.

2 In our preregistered analysis plan we intended to calculate Cronbach’s alpha for the items in the temporal distancing task to assess internal consistency. However, this is inappropriate as items in the temporal distancing task are randomized to different conditions for different people. The extent that a distress rating for a single item (e.g. “You fall over in the playground and everyone laughs at you”) correlates with the emotion ratings for all other items will depend on which condition it is in (no strategy vs. distant-future strategy).
